# First report of a *Plasmodium malariae* SSU rRNA gene variant in Africa associated with reduced amplification by nested PCR

**DOI:** 10.1186/s41182-025-00787-5

**Published:** 2025-08-04

**Authors:** Maki Goto, Kei Yamamoto, Kanako Komaki-Yasuda, Shigeyuki Kano, Norio Ohmagari

**Affiliations:** 1 Disease Control and Prevention Center, National Center for Global Health and Medicine, Japan Institute for Health Security (JIHS), 1-21-1 Toyama, Shinjuku-ku, Tokyo 162-8655 Japan; 2 Department of Tropical Medicine and Malaria, Research Institute of Global Health and Medicine, JIHS, 1-21-1 Toyama, Shinjuku-ku, Tokyo 162-8655 Japan

**Keywords:** *Plasmodium malariae*, Nested PCR, SSUrRNA, 18S rRNA, Species-specific primer, Sequence analysis

## Abstract

**Background:**

Nested polymerase chain reaction (PCR) targeting the small subunit ribosomal ribonucleic acid (SSU rRNA, 18S rRNA) region is widely used to differentiate *Plasmodium* species. We identified a variant of the *Plasmodium malariae* SSU rRNA gene that suggests nested PCR may fail to detect *P. malariae* strains with unknown mutations.

**Case presentation:**

A 56-year-old Japanese man developed a fever 2 months after returning from a 2-month stay in Sierra Leone. Quartan malaria was suspected based on blood smear findings, and nested PCR confirmed *P. malariae* infection. However, the secondary PCR band obtained using *P. malariae*-specific primers was fainter than the primary PCR band amplified with universal primers—a reversal of the typical pattern. Sequence analysis revealed a four-base deletion in the SSU rRNA gene within the primer-binding site of the species-specific reverse primer used in the secondary PCR, suggesting that mutations in this region may partially impair amplification and hinder species identification. *Cytochrome b* gene sequencing confirmed 100% identity with *P. malariae*.

**Conclusions:**

These findings underscore the need for continued molecular surveillance and sequence-based validation to ensure accurate diagnosis of *Plasmodium* infections, particularly in regions where genetic variants and zoonotic strains are emerging.

## Background

Precise identification of *Plasmodium* species and their variants is essential for effective malaria surveillance. Nested polymerase chain reaction (PCR) targeting the small subunit ribosomal RNA (SSU rRNA, 18S rRNA) gene is an established method for differentiating the six *Plasmodium* species: *P. malariae*, *P. falciparum*, *P. vivax*, *P. ovale curtisi*, *P. ovale wallikeri*, and *P. knowlesi*. In nested PCR, a secondary amplification is performed using species-specific primers following a primary PCR with universal primers. Typically, the secondary PCR yields fainter bands; however, amplification is still observed even when mutations are present within the species-specific primer-binding region [1]. Here, we report a case of a *P. malariae* SSU rRNA gene variant detected in Africa that exhibited reduced amplification in nested PCR.

## Case presentation

A 56-year-old Japanese man had spent 2 months in Freetown, Sierra Leone, for medical work. He had been prescribed doxycycline for malaria prophylaxis, but occasionally missed doses and discontinued it upon returning to Japan. He had not traveled abroad for at least 6 months before the onset of symptoms. Two months after his return, he developed recurrent nocturnal fevers that resolved spontaneously the following day. These episodes recurred five times at intervals of 12–48 h. On the 10th day after symptom onset, he presented to the hospital.

Upon arrival at the hospital, his body temperature was 37.9 °C, blood pressure 137/72 mmHg, and heart rate 95 bpm. Physical examination revealed no signs of jaundice in his ocular conjunctiva or hepatosplenomegaly. Blood tests showed the following: red blood cell count 4.72 × 10^5^/µL, hemoglobin 13.5 g/dL, platelet count 11.0 × 10^4^/µL, aspartate aminotransferase 34 IU/L, alanine aminotransferase 64 IU/L, and lactate dehydrogenase 311 IU/L. There was no evidence of hypoglycemia, metabolic acidosis, renal impairment, or hyperbilirubinemia. Additionally, a chest *X*-ray showed no abnormalities. Blood cultures showed no growth.

**Fig. 1:**
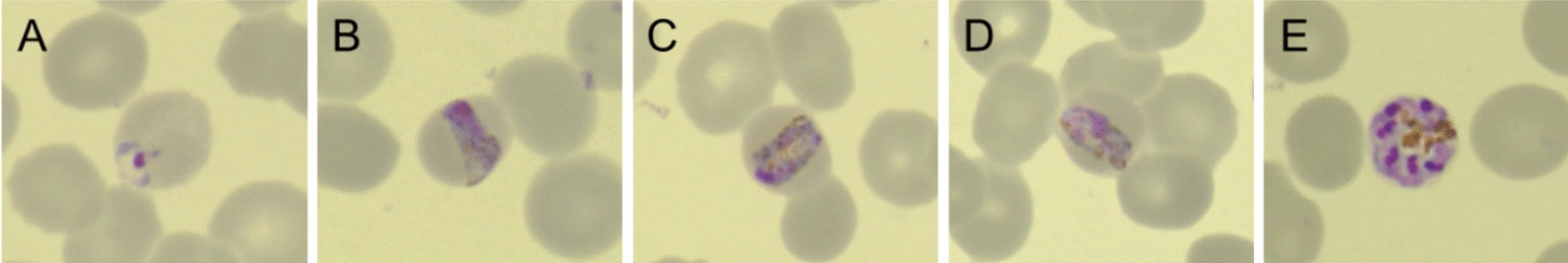
Microscopic images of Giemsa-stained thin-blood smears (× 1000 magnification). **A** Ring form; **B**–**D** band form; E schizont

A rapid diagnostic test (BinaxNow Malaria; Abbott) indicated non-falciparum malaria. Microscopic observation of Giemsa-stained thin-blood smears revealed parasitized erythrocytes with band-shaped trophozoites, characteristic of *P. malariae*, with a parasitemia of 0.059% (27.8 × 10^2^/µL) (Fig. 1).

The patient was diagnosed with uncomplicated quartan malaria and treated with artemether–lumefantrine for 3 days. He was discharged without complications after a fever clearance time of approximately 27 h and a parasite clearance time of approximately 36 h. These clearance times were within the expected range for artemether–lumefantrine treatment, indicating a favorable therapeutic response. He remained asymptomatic for 4 months after discharge.

**Fig. 2:**
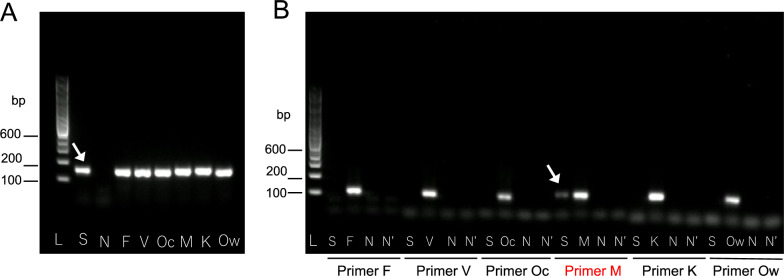
Results of nested polymerase chain reaction (PCR). **A** Primary PCR. Lane L: 100 bp DNA ladder. Lanes S, F, V, Oc, M, K, and Ow: the arrow indicates DNA templates from the patient’s blood (S) and positive controls containing partial small subunit ribosomal ribonucleic acid (SSU rRNA) gene sequences of *Plasmodium species, including P. falciparum* (F), *P. vivax* (V), *P. ovale curtisi* (Oc), *P. malariae* (M), *P. knowlesi* (K), and *P. ovale wallikeri* (Ow). Lane N: negative control (water); **B** secondary PCR. Lane L: 100 bp DNA ladder. Lanes S, F, V, Oc, M, K, and Ow: first-round PCR products using the same templates as in panel A. Lane N: first-round product from water. Lane N′: water-only control. The arrow indicates the PCR product amplified from the patient’s sample using *P. malariae*-specific primers (S). Primer sets used for each species are indicated below the gel

**Fig. 3:**
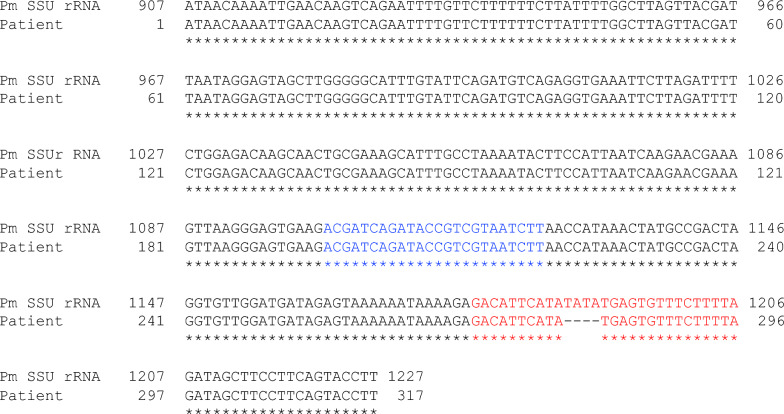
Partial sequence analysis of the small subunit ribosomal ribonucleic acid (SSU rRNA or 18S rRNA) (317 bp). A total of 372 base pairs (bp) were amplified using the originally designed forward primer (P0.5: CTGCGTTTGAATACTACAGCATGGA), located upstream of the primer used in the primary PCR (P1: ACGATCAGATACCGTCGTAATCTT [1, 2], shown in blue), and the reverse primer from the primary PCR (P2: GAACCCAAAGACTTTGATTTTTCTCAT [1, 2]). Of these, 317 bp were successfully sequenced. The 18S rRNA sequence of the present case was deposited to DDBJ/EMBL/GenBank under the accession number LC859598. This sequence, labeled as “Patient”, was aligned with the complete SSU rRNA sequence of *Plasmodium malariae*, labeled as “Pm SSU rRNA” (DDBJ/EMBL/GenBank accession number XR_003751927.1). In the patient’s DNA sequence, four bases corresponding to the *P. malariae*-specific reverse primer (indicated in red), used in nested PCR, were found to be deleted

**Fig. 4:**
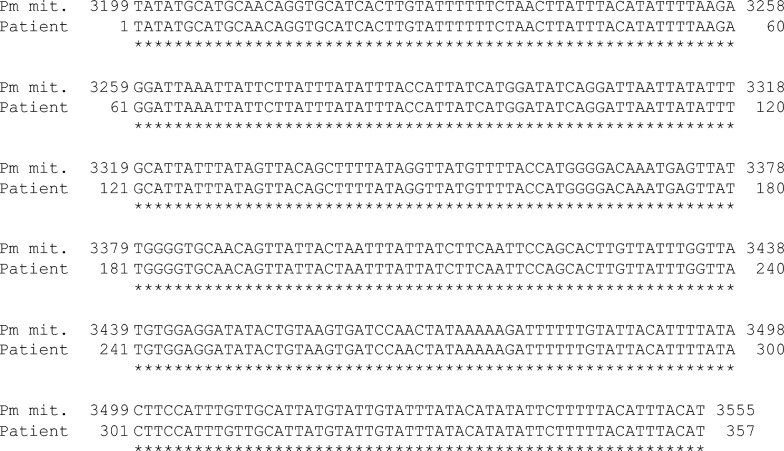
Partial sequence analysis of the *Cytochrome b* gene (357 bp). A 400-bp fragment amplified using two primers (RTPCRSc2_F: TGGAGTGGATGGTGTTTTAGA and Sc3R: ACCCTAAAGGATTTGTGCTACC [4]) yielded 357 bp of readable sequence. The data were deposited in DDBJ/EMBL/GenBank under the accession number LC859864. This sequence, labeled as “Patient”, was aligned with the complete mitochondrial DNA sequence of *Plasmodium malariae*, labeled as “Pm mit.” (DDBJ/EMBL/GenBank accession number LT594637.1). The patient’s sequence was 100% identical to the *cytochrome b* gene of *P. malariae*

Nested PCR was positive only for *P. malariae*, confirming species identification [1–3]. However, agarose gel electrophoresis showed that the secondary PCR band using *P. malariae*-specific primers was fainter than the primary PCR band obtained with universal primers (Fig. 2). Sequence analysis of the SSU rRNA gene revealed a four-base deletion at the binding site of the species-specific reverse primer (Fig. 3). *Cytochrome b* gene sequencing confirmed 100% identity with *P. malariae* (Fig. 4) [4].

## Discussion and conclusions

This case highlights an important diagnostic limitation of nested PCR: mutations in the primer-binding regions can reduce amplification efficiency and potentially lead to false-negative or ambiguous results. In standard nested PCR, secondary amplification typically yields stronger bands due to increased template specificity. However, in this case, the secondary PCR band using *P. malariae*-specific primers was unexpectedly fainter, despite confirmation of *P. malariae* infection through microscopy and *cytochrome b* gene sequencing. This suggests that even known species can carry undetected sequence variations that interfere with species-specific primer binding. When faint or absent bands are observed in the secondary PCR, sequencing of alternative gene regions should be considered to ensure accurate species identification.

Even when *P. malariae* is morphologically identified by microscopy, PCR testing should be performed, as well as sequencing and other genetic analyses, as appropriate. In the present case, despite observing the typical band formation of trophozoites in erythrocytes, it was challenging to estimate the parasite species accurately. Misidentification of *P. knowlesi* as *P. malariae* based on morphology has been previously reported, during the 2004 outbreak in Malaysia [5]. Furthermore, phylogenetically related *Plasmodium* species have also been found in African great apes, indicating zoonotic potential [6].

The variant of *P. malariae* observed in this patient was first detected in Africa, and according to the NCBI nucleotide collection database (https://www.ncbi.nlm.nih.gov/nucleotide), this variant has also been reported in Myanmar [DDBJ/EMBL/GenBank: AF487999.1] and Brazil [DDBJ/EMBL/GenBank: KJ619942.1]. Given that *P. malariae* can infect New World monkeys, chimpanzees, and gorillas, the variant seen in this case may be globally distributed across both human and non-human hosts [7].

In conclusion, surveillance using gene sequencing should be considered as a complementary tool to monitor the spread of unknown SSU rRNA gene variants in *Plasmodium* species. Such surveillance may facilitate ongoing assessment of the accuracy of nucleic acid testing and enable the early detection of emerging zoonotic threats. Although this study is based on a single case and does not presently undermine confidence in nested PCR assays, it nonetheless underscores the importance of accumulating additional data to confirm and validate these findings.

## Data Availability

The data supporting this case report are available from the corresponding author upon reasonable request.

## Abbreviations

PCR: Polymerase chain reaction
SSU rRNA: Small subunit ribosomal ribonucleic acid

## References

[1] Komaki-Yasuda K, Vincent JP, Nakatsu M, Kato Y, Ohmagari N, Kano S. A novel PCR-based system for the detection of four species of human malaria parasites and *Plasmodium knowlesi*. PLoS ONE. 2018;13: e0191886. 10.1371/journal.pone.0191886. 29370297 10.1371/journal.pone.0191886 PMC5785027

[2] Kimura M, Kaneko O, Liu Q, Zhou M, Kawamoto F, Wataya Y, et al. Identification of the four species of human malaria parasites by nested PCR that targets variant sequences in the small subunit rRNA gene. Parasitol Int. 1997;46:91–5. 10.1016/S1383-5769(97)00013-5.

[3] Canier L, Khim N, Kim S, Sluydts V, Heng S, Dourng D, et al. An innovative tool for moving malaria PCR detection of parasite reservoir into the field. Malar J. 2013;12:405. 10.1186/1475-2875-12-405. 24206649 10.1186/1475-2875-12-405 PMC3829804

[4] Goman M, Mons B, Scaife J. The complete sequence of a *Plasmodium malariae* SSU rRNA gene and its comparison to other plasmodial SSU rRNA genes. Mol Biochem Parasitol. 1991;45:281–8. 10.1016/0166-6851(91)90096-o. 2038360 10.1016/0166-6851(91)90096-o

[5] Baird JK. Malaria zoonoses. Travel Med Infect Dis. 2009;7:269–77. 10.1016/j.tmaid.2009.06.004. 19747661 10.1016/j.tmaid.2009.06.004

[6] Plenderleith LJ, Liu W, Li Y, Loy DE, Mollison E, Connell J, et al. Zoonotic origin of the human malaria parasite *Plasmodium malariae* from African apes. Nat Commun. 2022;13:1868. 10.1038/s41467-022-29306-4. 35387986 10.1038/s41467-022-29306-4 PMC8987028

[7] Collins WE, Jeffery GM. *Plasmodium malariae*: parasite and disease. Clin Microbiol Rev. 2007;20:579–92. 10.1128/CMR.00027-07. 17934075 10.1128/CMR.00027-07 PMC2176047

